# Interplay Between Diabetes Mellitus and the Occurrence of Osteoarthritis and Associated Conditions in Women of Menopausal Age

**DOI:** 10.7759/cureus.58502

**Published:** 2024-04-17

**Authors:** Rushikesh Shukla, Shailja Singh, Shruthi Kamath, Urmil Shah, Siddhi Patel, Krish Kherajani, Ananya Gupta, Priya Shaw, Vishnu Unnithan, Sharel Kaithathara, Pankaj Gharde

**Affiliations:** 1 Medicine, Jawaharlal Nehru Medical College, Datta Meghe Institute of Higher Education and Research, Wardha, IND; 2 Medicine, Kasturba Medical College, Mangalore, Mangalore, IND; 3 Medicine, Rajiv Gandhi Medical College, Thane, IND; 4 Medicine, Dr. D. Y. Patil Medical College, Hospital and Research Centre, Pune, IND; 5 Medicine, Terna Medical College, Navi Mumbai, IND; 6 Medicine, Burdwan Medical College and Hospital, Bardhaman, IND; 7 Nuclear Medicine, King Edward Memorial Hospital and Seth Gordhandas Sunderdas Medical College, Mumbai, IND; 8 Rheumatology, New Vision University, Tbilisi, GEO; 9 General Surgery, Jawaharlal Nehru Medical College, Datta Meghe Institute of Higher Education and Research, Wardha, IND

**Keywords:** obesity, metabolic disease, menopause, type 2 diabetes, osteoarthritis

## Abstract

Osteoarthritis (OA) and diabetes mellitus (DM) have long-term deleterious chronic effects and are among the most prevalent chronic disorders. DM and its associated factors, such as hyperglycemia, have a significant contribution to the pathophysiology of OA, particularly in post-menopausal women. Women who have uncontrolled diabetes (DM) are more prone to develop osteoarthritis (OA), which may be exacerbated by poor glycemic control. Furthermore, this category of female patients with DM has an increased risk of developing fractures, even in those with initially normal bone density scores, further illustrating the correlation between DM and bone health. Additionally, multiple risk factors, including obesity, metabolic syndrome, hypertension, estrogen-based hormone therapy, and hyperuricemia, in menopausal women can lead to the development and exacerbation of OA. It is discovered that these variables have a direct or indirect impact, frequently causing inflammation and hormonal changes, which contribute to the intricate interaction between DM and OA. The management of OA and DM in women thus calls for a multi-faceted management plan including glycemic control, weight control, exercise, and specialized pain management methods catering to the specific requirements of the patients. Regularly screening for OA should be implemented for menopausal women with DM and utmost care should be provided by healthcare professionals. Regular monitoring of joint health and early management, encouraging interdisciplinary cooperation, putting preventative measures into place, and creating individualized treatment programs are essential. A thorough understanding of the link between DM and OA will ultimately lead to improved health outcomes and a better future for these individuals.

## Introduction and background

**Figure 1:**
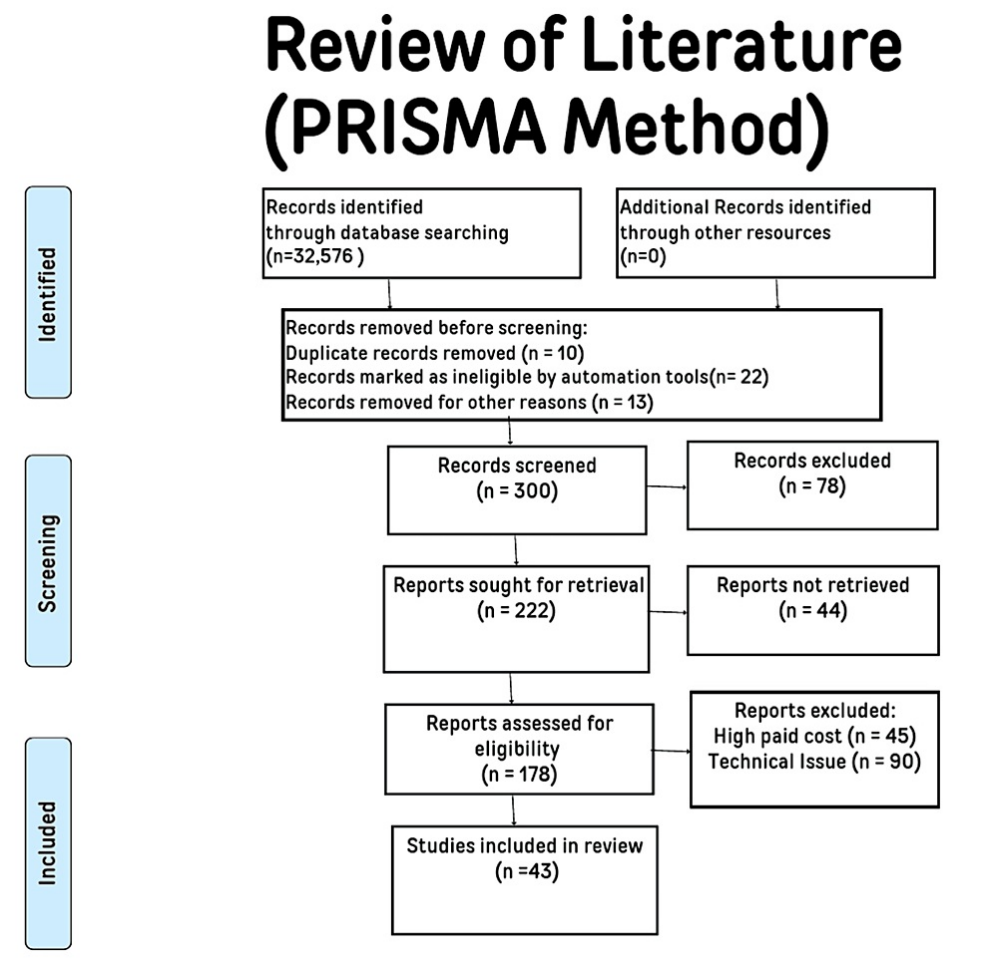
PRISMA search strategy for this review PRISMA: Preferred Reporting Items for Systematic Reviews and Meta-Analyses

Diabetes mellitus (DM) is a globally prevalent chronic illness. By 2030, the prevalence of this illness among adults is expected to increase substantially from 6.4% in 2010 to 7.7% [1]. The heart, kidneys, eyes, and other organs sustain significant damage. The most prevalent kind of rheumatic illness and a significant contributor to disability is osteoarthritis (OA); over 10% of people worldwide suffer from OA [2]. OA sufferers' ability to move about is hindered by severe joint discomfort. Recent findings indicate that OA is a metabolic illness linked to metabolic syndrome [3]. For the study, the following databases were used: PubMed/MEDLINE, Embase, Science Direct, Cochrane Library, and the Clinical Trials site for papers published up to September 2023. Electronic and manual data sources were searched. Excluded from the search results were studies conducted in languages other than English, as well as duplicate reports, those incurring high costs, those flagged by automation tools as ineligible, those presenting technical issues, and those that were unretrievable. The phrases “osteoarthritis” [All Fields] AND “menopausal age women” [MeSH phrases] OR “diabetes mellitus” [All Fields] AND “menopausal women” [MeSH term] were used in PubMed/MEDLINE literature search. The words mentioned above (interplay between DM and the occurrence of OA in women at menopausal age) were employed in the search strategy on the Cochrane Library, the database for systematic review. We used a qualitative method to study the data and focused on identifying repeating themes and patterns that emerged from the studies included in our research. Figure 1 shows the Preferred Reporting Items for Systematic Reviews and Meta-Analyses (PRISMA) method flowchart.

## Review

**Figure 2:**
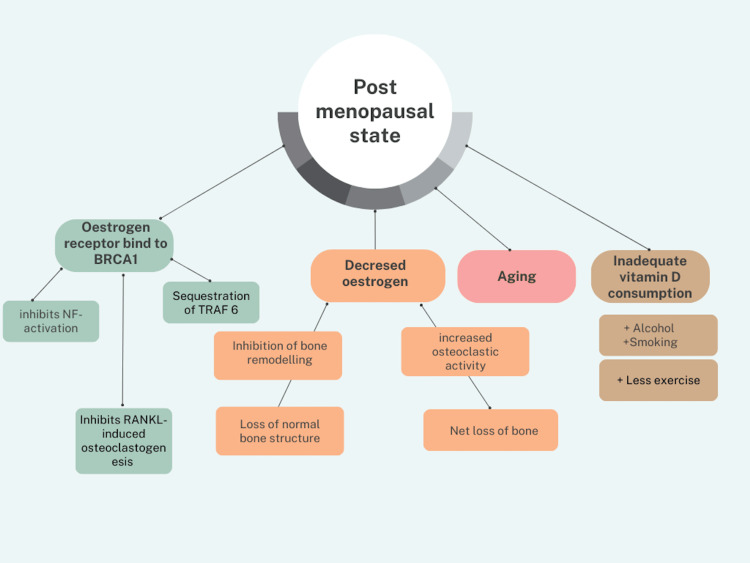
Factors in post-menopausal women leading to increased risk of osteoporosis BRCA1: Breast cancer 1; TRAF6: Tumor necrosis factor receptor (TNFR)-associated factor 6; RANKL: Receptor activator of nuclear factor-κB-ligand; NF: Nuclear factor The figure is the author's own creation.

OA and DM both exhibit enduring, harmful chronic impacts and rank among the most commonly occurring chronic conditions. OA is among the significant reasons for the loss of economy due to disability among women, with the majority of OA patients facing limitations and restrictions in performing daily activities and work. Among the various long-term effects of diabetes, including macro and microvascular pathologies that can lead to heart attacks and strokes, further contributing to disability and morbidity, research indicates that individuals with diabetes had a 29.5% prevalence of OA, while patients without diabetes had a 14.4% prevalence of OA [4]. The connection between the two diseases is also supported by the fact that it has been discovered that type 2 diabetes mellitus (T2DM) is a risk factor for OA and independently predicts the likelihood of requiring complete joint replacement [5]. It is crucial to understand how DM and OA tie in with the increasing age of women and the changes that are often associated with age advancement. Osteoporosis (OP) is a skeletal condition typified by diminished bone mass and bone mineral density (BMD), concomitant with the degradation of bone microarchitecture, heightened bone fragility, and susceptibility to fractures. OA can be distinguished by the progressive deterioration of articular cartilage, inflammatory responses within the joint, and alterations in periarticular and subchondral bone structures [6]. OP, which refers to the net loss of bone mass, not to be confused with OA which is a disease causing reduced joint mobility, function, and pain, is another disease closely associated with DM that contributes to morbidity and mortality among post-menopausal women. This is due to a drop in estrogen levels after menopause, which prevents the hormone from performing its primary actions like bone remodeling, thus leading to loss of normal bone architecture. Additionally, there is an increased osteoclastic activity due to the post-menopausal activation of osteoclasts by lack of estrogen. The estrogen receptor/breast cancer anti-estrogen resistance 1 (ER/BCAR1) complex is also created when the estrogen receptor binds to breast cancer 1 (BRCA1). This combination inhibits nuclear factor (NF)-activation and receptor activator of nuclear factor-κB-ligand (RANKL)-induced osteoclastogenesis while sequestering tumor necrosis factor receptor (TNFR)-associated factor 6 (TRAF6). Aging, less exercise, inadequate vitamin D consumption, and alcohol and cigarette use impair bone mineral density, resulting in a lower T-score and raising the risk of OP [6-8]. Figure 2 shows all the factors of post-menopausal women that lead to an increased risk of OP.

A study conducted in Beijing, China from 2015 to 2017 revealed that out of 1,046,264 adult patients diagnosed with T2DM, 32.65% also had OA, of which 38.05% were women [9]. Another study conducted on 420 Bahraini women aged 50-59 years diagnosed with OA revealed that 43% had a combination of various comorbidities, out of which 10% reported a history of diabetes [10]. In contrast to DM, which affects 12% of adults aged 20 and up and 26% of those aged 65 and up, OA is a common disease that impacts 14% of adults aged 25 and up and 34% of those aged 65 and up. Roughly 40% of individuals with OA complain of arthritis-associated restrictions in everyday activities, and 30% face a problem with work-related tasks, making OA the leading source of disability and financial burden. About 12% of individuals over 20 and 26% over 65 have diabetes [11]. There were 341,561 instances of OA among individuals with T2DM, with a prevalence of 32.65%. Additionally, there were 38.05% more females than boys overall, and this disparity was significant statistically. The incidence of OA was present in all age categories within the group of patients with T2DM; the age group of 65-69 years showed to have the greatest prevalence (36.76%), and the age group of 44 years was noticed to have the lowest prevalence (14.3%). Prior to turning 70, the frequency rose with age [5]. The unrefined incidence of arthritis was 48.1%, which is 9.6 million among people with DM in the most recent research conducted in the USA, without considering other factors. After adjusting for additional variables, the reported prevalence of arthritis and arthritis-related activity limitation (AAAL) among arthritic persons with DM compared to people without DM were 1.44 (1.35 - 1.52) and 1.21 (1.15 - 1.28), respectively [12]. In a research study, 110 post-menopausal women took part. Of these, 55 women with T2DM showed early symptoms of OA, including morning rigidity lasting longer than 30 minutes and soreness. Fasting blood sugar (FBS), hemoglobin A1c (HbA1c), and lipid levels, including means for total cholesterol (TC), triglycerides (TG), very low-density lipoprotein cholesterol (VLDL-C), and low-density lipoprotein cholesterol (LDL-C), were considerably higher, while good cholesterol high-density lipoprotein cholesterol (HDL-C) was notably lower [13]. It was found that for the menopausal hormonal therapy group compared to the non-menopausal hormonal therapy group, the odds ratio for OA was 0.70 [14]. A women's health initiative-based study including 93,000 post-menopausal women revealed that, despite having initially normal BMD scores, women with T2DM had a 20% greater risk of fracture than those without the disease [15]. Higher BMD values of 0.045 in the post-menopausal women group at the spinal site of diabetic patients than in patients without diabetes were found in research to determine the relationship between bone mass and T2DM in women of Iran. Numerous variables, including theobromine use, UV exposure, delayed menarche, very little to no exercise, smoking habits, heredity, and, most significantly, poor calcium intake, are risk factors for OP. While the frequency of OP was not statistically significant, the widespread presence of early menopause was greater in women diagnosed with diabetes (P=0.046). Post-menopausal women diagnosed with diabetes were more commonly affected by OP (41.6%) than diabetic premenopausal women (7%). However, the prevalence of OP was 15% in non-diabetic premenopausal women and 36.8% in non-diabetic post-menopausal women [16]. Table 1 summarizes the key insights from all the studies mentioned above.

**Table 1:** Brief summary of key insights of mentioned studies OA: Osteoarthritis; DM: Diabetes mellitus; FBS: Fasting blood sugar; HbA1c: Hemoglobin A1c; BMD: Bone mineral density; T2DM: Type 2 diabetes mellitus

Study/Association	Findings
Study in Beijing, China (2015-2017) [6]	32.65% of T2DM patients had OA; 38.05% of OA patients were women.
Study on Bahraini women (50-59 years) [7]	43% of OA patients had comorbidities; 10% reported a history of diabetes.
Study by Cheng et al. on the prevalence of diagnosed arthritis and arthritis-related activity limitation [8]	DM affects 12% of adults aged 20+ and 26% aged 65+; OA impacts 14% of adults aged 25+ and 34% aged 65+; OA is a leading source of disability.
Research in the USA by Jung et al. [9]	48.1% incidence of arthritis in people with DM; Adjusted prevalence ratios for arthritis and activity limitation were 1.44 and 1.21.
Study on post-menopausal women with DM by Malot et al. [10]	Women with T2DM showed early OA symptoms; Higher FBS, HbA1c, and lipid levels were observed.
WHI-based study on post-menopausal women by Khadilkar [12]	Women with T2DM had a 20% greater risk of fracture.
Research on BMD in Iranian women by Maghbooli et al. [13]	Higher BMD in post-menopausal diabetic women; Osteoporosis is more common in diabetic women.

The above studies reveal that there is a significant association present between T2DM and OA. It is highlighted that OA often exists with other underlying or overt health issues. This is supported by the fact that patients with T2DM also exhibit early OA symptoms, potentially linked to blood sugar control. Additionally, the risk of OA increases with age among T2DM, and those with both conditions are a victim of facing substantial activity limitations hampering their daily life. Moreover, women with T2DM have a higher risk of fractures and OP, emphasizing a connection between T2DM and reduced bone density, particularly in post-menopausal women. It is also imperative to focus on associations established by researchers for the occurrence of OA in women with other systemic conditions to fully comprehend the potential underlying pathology. A study by Asokan et al. suggested that 10% of those with a history of diabetes also had an OA diagnosis [7]. Post-menopausal women also have a higher risk of developing T2DM [10]. It has also been significantly established that hyperglycemia contributes to bone degradation and could be the culprit for OA. In prospective research done in a tertiary care hospital in Rajasthan, it was discovered that diabetic post-menopausal women with OA had significantly higher HbA1c and FBS levels than the control group, which further supports the link between diabetes and OA [10].

Cardiovascular conditions

Circulating sex hormones affect adiposity levels through pathways including inflammation and endothelial dysfunction, which connect OA with cardiovascular disease (CVD) illness [14,15]. Acute and chronic inflammation that leads to joint degeneration harms the arteries that supply bones and promotes CVD-related events. Due to the loss of estrogen's musculoskeletal-protective properties with menopause, post-menopausal women have a greater prevalence of OA and CVD [14]. It is necessary to consider the influence of environmental and socioeconomic elements that contribute to disease, in addition to overlapping biological and physiological components. DM, hence, in turn, can itself become an independent risk factor for developing OA and CVD individually, which also serves as a potential bridging factor connecting OA and DM in post-menopausal women.

Metabolic conditions

Type 2 Diabetes

Compared to people without T2DM, those with it have a greater incidence of arthritis [10,13]. A study by Hart et al. found a directly proportional relationship between blood sugar and unilateral and bilateral knee OA in women aged 45 to 64 [16,17]. Post-menopausal women with T2DM have symptoms of menopause, such as joint pain, that are more severe with poor glucose management [18]. Another study indicated that per particular research, 5,788 patients with DM had an overall OA prevalence of 29.51%, while 645,088 patients with OA had a typical DM prevalence of 14.41% [19]. People suffering from DM had a higher probability of developing OA complications than individuals without DM. The frequency of DM in the OA category was 9.7% in Rahman et al. study [19] and 9.8% in Wang et al. work, both taken from a Congress report [20]. Therefore, it can be said that poorly controlled diabetes can be a reason for developing OA in women.

Obesity and Metabolic Syndrome

Studies conducted on various female age groups nearing menopausal/perimenopausal age focusing on causes of OA and bone-associated problems described obesity as one of the factors that could precipitate the condition [21]. In a study conducted among 420 women in Bahrain ranging from the age group 50-59 years [21], 74% of patients had significant joint involvement, 21% had generalized OA, and 5% involved small joints. Half of the women surveyed were obese and 30% were overweight with the multivariate analysis demonstrating significant variables to be age, body mass index (BMI), menopausal status, comorbidities, and family history [7]. A status of overweight, grade I/II obesities increased the chance of developing knee OA by 2-4.7 folds [22,23]. For example, in this case, patients with grade II obesity had incidence rates of knee OA five times greater than those with an average weight. Obesity is associated with elevated plasma leptin levels [10], and the same hormone is found abundantly in the synovial fluid of OA-affected joints [24,25].

In a pilot study on a comparative basis with 25 participants with knee OA and 19 without knee OA, 80% of participants with knee OA were found to be diagnosed with metabolic syndrome (MetS), while only 26% among the participants without knee OA [24]. The difference in the MetS prevalence was significant between the two groups, and each factor of MetS was significant in the OA group [24]. One hypothesis for this is related to mitochondrial dysfunction over signaling pathways and disturbances in the cartilage production and repair mechanism [25]. The efflux function of cartilage may be compromised when cholesterol builds up in the tissue, leading to the development of OA [26,27]. After aging, the second most common kind of OA might now be metabolic OA [22]. Therefore, MetS can also be associated as a risk factor for the pathophysiology of OA.

Hypertension

OA and hypertension (HTN) have similar risk variables, such as age, obesity, and sex [28]. These risk factors and long-term inflammation may be the underlying cause of the link between OA and HTN. A critical factor in the connection between HTN and OA, particularly knee OA, is interleukin 6, one of the most significant pro-inflammatory cytokines [29,30]. Inoue et al. studied community participants aged 30-86 years, and the prevalence of hypertension in the OA group was higher in both men and women, but the relationship with MetS was found only in women. These results suggest that the relationship between OA and HTN was also raised in premenopausal women [14]. Additionally, mutations in the vitamin D receptor were demonstrated to have contributed to a tenable pathway connecting OA with HTN [31,32].

Estrogen-based hormone therapy

Studies have been done on a demographic mainly focusing on menopausal and post-menopausal women taking estrogen-based hormone therapy (HT) to establish a relation between HT and OA; some studies did conclude that estrogen-based therapy had a protective role in the occurrence of OA, given its lower incidence or prevalence on women who received HT [2,33-38]. In contrast, others found no relevant relation between the two and suggested age and weight were more statistically significant associations with OA [16]. Although some studies suggest relations between the two, there are only a few studies that have been conducted as randomized clinical trials from an interventional perspective for OA [39]. Many studies and clinical trials are still underway to be conducted on a larger scale so that more significant data can be acquired to get concrete evidence of the effect of HT on the cases of OA, whether a negative association or a positive one.

Hyperuricemia

Association between gout and menopause has been established by a particular study revealing that gout was discovered after menopause started in 21 out of 23 women (91%). Six women (26%) had tophaceous gout, and 13 women (57%) had polyarticular involvement; 70% of cases had an underlying arthropathy, often OA. Gout is common in women, especially post-menopausal women who are either on diuretic treatment or have renal insufficiency [40]. Nested case-control research conducted by two general practices in Nottingham found that, after controlling for risk variables, the presence of clinically diagnosed OA was significantly associated with individual joint site gouty episodes [41]. There is persistent OA discomfort in joints afflicted by gouty arthritis [42] with people afflicted by gout having a greater frequency of knee OA than those without gout [43]. Table 2 summarises the studies mentioned in the text with their findings.

**Table 2:** Brief summary of the associations established by researchers between OA and different conditions OA: Osteoarthritis; DM: Diabetes mellitus; FBS: Fasting blood sugar; HbA1c: Hemoglobin A1c

Study/ Association	Findings
Malot et al. [10]	Diabetic post-menopausal women with OA had significantly higher HbA1c and FBS levels than the control group.
Hart et al. [11]	A link between blood sugar as well as unilateral and bilateral knee OA in women aged 45 to 64.
Rouen et al. [18]	Post-menopausal women with type 2 diabetes have symptoms of menopause that are more severe with poor glucose management.
Rahman et al. [19]	5788 patients with DM had an overall OA prevalence of 29.51%, while 645,088 patients with OA had a typical DM prevalence of 14.41%.
Wang et al. [20]	The frequency of DM in the OA category was 9.8%.
Asokan et al. [7]	74% had major joint involvement in OA; 21% had generalized OA; 5% involved small joints; Half of the women were obese and 30% were overweight.
Nemet et al. [24]	80% of participants with knee OA were found to be diagnosed with metabolic syndrome, while only 26% of the participants without knee OA.

In summary, the connection between menopausal women, DM, OA, and various metabolic conditions is multifaceted. The systemic conditions mentioned can act as a potential risk factor or a direct link through an interplay of hormones and hormonal changes, inflammation, and other factors for the development of OA in diabetic women. The relationship between diabetes and OA is further compounded by other conditions such as CVDs, HTN, obesity, and hyperuricemia. These comorbidities often coexist with diabetes, with perimenopausal and menopausal women being at a higher risk of acquiring these diseases.

## Conclusions

The interplay between OA and DM has long-drawn chronic effects and is among the most prevalent chronic disorders. The coexistence of OA and DM in females presents a complex and multifaceted challenge that highlights the necessity of all-encompassing management plans. T2DM has an effect on OA that goes beyond simple risk factor correlation. Research has uncovered plausible biochemical routes, such as modified glucose metabolism and AGE products, that could aggravate the symptoms of OA in people with diabetes and lead to joint tissue destruction. Underlying pathologies and systemic conditions that can lead to exacerbation of both conditions, like obesity, hyperuricemia, and cardiovascular diseases, along with socioeconomic and environmental factors, also need to be addressed. Taken together, studies have been showing that comprehensive and integrated strategies must be followed aiming to provide glycemic control, weight- and pain-management techniques, and physical activity to prevent and treat OA". Furthermore, because females with DM are more susceptible to OA, healthcare practitioners should place a high priority on routine monitoring and assessment of joint health in these patients. They should also act quickly to address any symptoms or difficulties. In conclusion, OA in women with DM is a challenging clinical situation that calls for an integrative approach. Through the promotion of interprofessional collaboration, the implementation of preventative measures, and the development of customized treatment plans, the goal should be to enhance the quality of life for these individuals and lessen the impact of these interconnected conditions, which will improve the general well-being and health outcomes of impacted women and lead to a better future for them.
